# UNDERSTANDING CHALLENGES IN DEMENTIA CARE AMONG AFRICAN AMERICAN AND LATINX POPULATIONS

**DOI:** 10.1093/geroni/igad104.2419

**Published:** 2023-12-21

**Authors:** Rifat Alam, Brianna Sirdich, Andiara Schwingel

**Affiliations:** University of Illinois at Urbana-Champaign, Urbana, Illinois, United States; University of Illinois at Urbana-Champaign, Champaign, Illinois, United States; University of Illinois, Urbana Champaign, Champaign, Illinois, United States

## Abstract

Addressing disparities in dementia care among minoritized populations in the US is obligatory. This qualitative study explores perspectives of healthcare providers and administrators about challenges in delivering dementia care among African American and Latinx populations. 12 healthcare providers and administrators from 5 organizations in Illinois and Indiana were interviewed virtually between October 4, 2022, and March 7, 2023, and transcribed verbatim. Interviews were coded and three emerging themes were identified inductively describing participants’ perspectives. Delays in treatment-seeking: Includes challenges of the communities to understand normal aging and dementia and trust healthcare system, lack of knowledge of available resources and perceived cost, tradition of informal caregiving and diminished insights of patients. Lack of a comprehensive support system: Describes areas hindering providers and administrators becoming a support for the patients, includes- communication barriers to understand language and vernaculars, lack of access to healthcare, insurance, and resources by the minoritized communities and needs for an efficient primary healthcare team. Difficulties in patient evaluation: Time constraints of the primary care providers and shortage of specialists affect proper evaluation of patients. Engaging patients from low-income groups and immigrants in the system is complex. Non-compliance due to unavailability of transportation and telemedicine and comorbidities of older patients leads to a failure of complete evaluation of patients’ cognitive function that needs multiple visits. Our study highlights prevailing challenges and areas of advancement of dementia care among African American and Latinx populations. Developing a roadmap for a policy-level change and cross-culturally valid interventions will help address dementia inequities.

